# Primarily Disrupted Default Subsystems Cause Impairments in Inter-system Interactions and a Higher Regulatory Burden in Alzheimer's Disease

**DOI:** 10.3389/fnagi.2020.593648

**Published:** 2020-11-11

**Authors:** Huihui Qi, Yang Hu, Yingru Lv, Peijun Wang

**Affiliations:** ^1^Department of Medical Imaging, Tongji Hospital, Tongji University School of Medicine, Tongji University, Shanghai, China; ^2^Laboratory of Psychological Health and Imaging, Shanghai Mental Health Center, Shanghai Jiao Tong University School of Medicine, Shanghai, China; ^3^Department of Imaging, Huashan Hospital, Fudan University, Shanghai, China; ^4^Department of Medical Imaging, Tongji Hospital Affiliated With Tongji University, Shanghai, China

**Keywords:** Alzheimer's disease, dynamic effective connectivity, resting-state functional MRI, large-scale networks, Granger causality analysis, subsystem

## Abstract

**Background:** Intrinsically organized large-scale brain networks and their interactions support complex cognitive function. Investigations suggest that the default network (DN) is the earliest disrupted network and that the frontoparietal control network (FPCN) and dorsal attention network (DAN) are subsequently impaired in Alzheimer's disease (AD). These large-scale networks comprise different subsystems (DN: medial temporal lobe (MTL), dorsomedial prefrontal cortex (DM) subsystems and a Core; FPCN: FPCNA and FPCNB). Our previous research has indicated that different DN subsystems are not equally damaged in AD. However, changes in the patterns of interactions among these large-scale network subsystems and the underlying cause of the alterations in AD remain unclear. We hypothesized that disrupted DN subsystems cause specific impairments in inter-system interactions and a higher regulatory burden for the FPCNA.

**Method:** To test this hypothesis, Granger causality analysis (GCA) was performed to explore effective functional connectivity (FC) pattern of these networks. The regional information flow strength (IFS) was calculated and compared across groups to explore changes in the subsystems and their inter-system interactions and the relationship between them. To investigate specific inter-system changes, we summed the inter-system IFS and performed correlation analyses of the bidirectional inter-system IFS, which was compared across groups. Additionally, correlation analyses of dynamic effective FC patterns were performed to reveal alterations in the temporal co-evolution of sets of inter-subsystem interactions. Furthermore, we used partial correlation analysis to quantify the FPCN's regulatory effects. Finally, we applied a support vector machine (SVM) linear classifier to probe which network most effectively discriminated patients from controls.

**Results:** Compared with controls, AD patients showed a decreased intra-DN regional IFS, which was significantly related to the inter-network's IFS. The IFS between the DN subsystems and FPCN subsystems/DAN decreased. Critically, the correlation values of the decreased bidirectional IFS between the DN subsystems and FPCNA diminished. Additionally, the Core and DM play pivotal roles in disordered temporal co-evolution. Furthermore, the FPCNA showed enhanced regulation of the Core. Finally, the MTL subsystem and Core were effective at discriminating patients from controls.

**Conclusion:** The predominantly disrupted DN subsystems caused impaired inter-system interactions and created a higher regulatory burden for the FPCNA.

## Introduction

Resting-state functional magnetic resonance imaging (rs-fMRI) has emerged as a powerful, non-invasive tool for measuring temporal correlations in spontaneous blood oxygen level-dependent (BOLD) signal fluctuations in discrete brain regions. Spatially distributed brain regions within neuroanatomical systems spontaneously activate in concert as large-scale networks, thereby delineating the functional network architecture of the human brain (Allen et al., 2011; Power et al., 2011; Van den Heuvel and Sporns, 2013). Recent studies have witnessed extraordinary interest in the default network (DN), dorsal attention network (DAN), and frontoparietal control network (FPCN) since these resting state networks (RSNs) are reproducible and not only reflect human brain intrinsic structure and function but also provide potential sensitive hallmarks for disease processes (Greicius et al., 2004; Damoiseaux et al., 2006; Sorg et al., 2007; Liao et al., 2010; Yeo et al., 2011; Wang et al., 2015; Zhan et al., 2016). The DN showed increased activity during rest and tasks involving aspects of self-generated thought (Andrews-Hanna et al., 2014). In contrast, the DAN showed increased activation during cognitive tasks that require externally focused visuospatial attention (Fox et al., 2005; Dosenbach et al., 2007). The FPCN, as a regulatory network, mediates the dynamic balance between the DN and DAN by flexibly coupling its activity with one or the other, and thereby driving either internally or externally directed cognition, respectively (Gao and Lin, 2012; Spreng et al., 2013; Elton and Gao, 2014). Alzheimer's disease (AD) is a progressive neurodegenerative disease characterized by early predominant memory impairment and subsequent disturbances in other cognitive functions with progression of the disease (Krajcovicova et al., 2014). Corresponding with the clinical symptoms, studies have found that the memory-related DN was first disrupted in AD (Greicius et al., 2004); however, other networks have since been implicated, including those involved in visuospatial and executive function networks (Brier et al., 2012; Jones et al., 2015). Recently, converging evidence has revealed that these large-scale networks are not a unitary entity but can be divided into distinct subsystems associated with more specific cognitive processes (Buckner et al., 2008; Andrews-Hanna et al., 2010; Kim, 2012; Salomon et al., 2014; Dixon et al., 2018). Andrews-Hanna et al. (2010), using hierarchical clustering techniques, revealed that the DN comprised three distinct subsystems: a dorsomedial prefrontal cortex (DM) subsystem that is preferentially activated during the generation of inferences about one's present situation or mental state, a medial temporal lobe (MTL) subsystem that is selectively involved in self-relevant predictions about one's future and recollection of episodic memories, and a Core that supports self-referential processing (Andrews-Hanna et al., 2010, 2014). Dixon et al. (2018) identified two distinct subsystems within the FPCN based on hierarchical clustering and machine learning classification analyses of within-FPCN functional connectivity (FC) patterns and found that the FPCNA was preferentially coupled with the DN, which was mainly involved in the regulation of introspective processes; on the other hand, the FPCNB exhibited stronger connectivity with the DAN, which was preferentially involved in the regulation of visuospatial perceptual attention. Recent studies have indicated different levels of disruption in distinct subsystems of the DN in AD (Buckner et al., 2005; Zhang et al., 2010; Jones et al., 2011; Qi et al., 2018). However, little is known about changes in the causal interactions among the subsystems of these large-scale brain networks and the underlying causes of these alterations in AD. Based on the above findings, we envisaged that significantly disrupted DN subsystems initiated their specifically disordered interactions with FPCN subsystems and the DAN, and placed a higher processing burden on the FPCNA. To test our hypotheses in this study, Granger causality analysis (GCA) was used to construct effective connectivity networks for 27 normal controls (NCs) and 24 patients with mild to moderate AD (Granger, 1969). GCA is a method to infer directional influences among brain regions and has been proven to be effective in investigating the causal networks according to previous neuroimaging studies (Winston, 2013; Zhong et al., 2014). GCA is data-driven and rests upon multivariate or vector autoregressive models for functional MRI time-series to test for directed connections and can provide information about the dynamics and directionality of the fMRI BOLD signal in cortical circuits (Harrison et al., 2003; Zhou et al., 2009). We performed regional information flow strength (IFS) analyses to identify the prominently disrupted systems and changes in their causal interactions with other systems as well as the relationships between them. Then, the inter-system effective connectivity analyses were used to further explore specific changes in the inter-system causal interactions in AD. Additionally, we performed correlation analyses of inter-system dynamic effective connectivity to explore alterations in the temporal co-evolution of sets of inter-subsystem interactions. Furthermore, we quantified the regulatory effects of FPCNA on DN subsystems and inter-system interactions. Finally, a support vector machine (SVM) linear classifier was performed to probe which network contributed the most to the discrimination between AD patients and NCs.

## Materials and Methods

### Participants

A total of 55 subjects were enrolled from Shanghai Huashan Hospital. All participants were categorized into an NC group (*n* = 27) and an AD patient group (*n* = 28). AD patients were diagnosed by a qualified neurologist using criteria for amnestic AD (Fennema-Notestine et al., 2009) and had mini-mental state examination (MMSE) scores between 12 and 27 (inclusive) and clinical dementia rating (CDR) scores of 1 or 2. The control groups had MMSE scores between 26 and 30 (inclusive) and CDR scores of 0. The data of four subjects (four patients with AD) were excluded due to excessive motion, severe brain atrophy, hydrocephalus or large areas of cerebral infarction. Details regarding the clinical and demographic data of the remaining 51 subjects are shown in Table 1. There were no significant differences in terms of sex or age between the two groups.

**Table 1:** Demographics and clinical information.

**Characteristics**	**NC (*n* = 27)**	**AD (*n* = 24)**	***P***
Age	63.74 ± 7.80	67.54 ± 10.48	0.146^a^
Female/Male	11/16	13/11	0.406^b^
MMSE	28.84 ± 1.19	21.46 ± 1.67	<0.001^a^

*Data are presented as the mean ± standard deviation (SD)*.

a *The P-value was obtained using a two-sample t-test*.

b *The P-value was obtained using the Pearson chi-square test*.

### Image Acquisition and Pre-processing

All subjects underwent whole-brain MRI scanning with a 3.0-T SIEMENS Verio scanner. Resting-state BOLD functional MRI data were collected using an echo-planar imaging (EPI) sequence. The scanning parameters were TR = 2,000 ms, TE = 35 ms, FOV = 25.6 × 25.6 cm, flip angle = 90, matrix = 256 × 256, slices = 33, thickness = 4 mm, and gap = 4 mm. Unless specifically stated otherwise, all of the pre-processing was performed using statistical parametric mapping (SPM8, http://www.fil.ion.ucl.ac.uk/spm). The first ten images were discarded in consideration of magnetization equilibrium. The remaining 190 images were corrected for the acquisition time delay among different slices. Then, the images were realigned to the first volume for head-motion correction. The fMRI images were further spatially normalized to the Montreal Neurological Institute (MNI) EPI template and resampled to a 3-mm cubic voxel. Several sources of spurious variance, including the estimated motion parameters, the linear drift, and the average time series in the cerebrospinal fluid and white matter regions, were removed from the data through linear regression. Temporal bandpass filtering (0.01–0.08 Hz) was performed to reduce the effects of low-frequency drift and high-frequency noise. Finally, we conducted spatial smoothing with a Gaussian kernel (full-width-at-half-maximum [FWHM] 6 mm). The time course of head motion was obtained by estimating the translations in each direction and the rotations in angular motion about each axis for each of the 155 consecutive volumes. All the subjects included in this study exhibited a maximum displacement of <3 mm (smaller than the size of a voxel in a plane) at each axis and an angular motion of <3 degrees for each axis. Data from two subjects were excluded due to excessive motion.

### Region of Interest Definition

**Figure 1:**
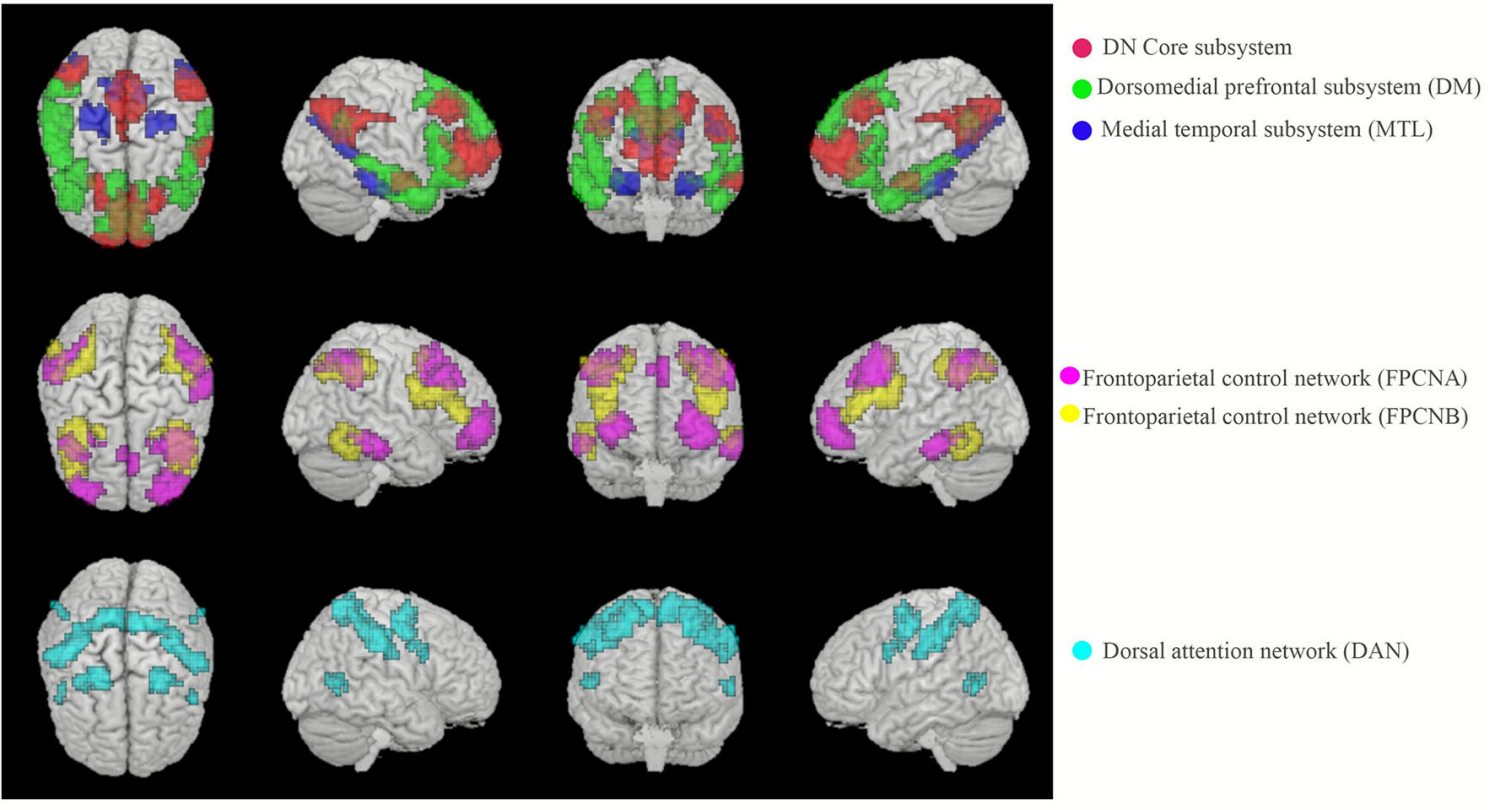
ROIs of the MTL subsystem, DM subsystem, Core, FPCNA subsystem, FPCNB subsystem, and DAN.

We selected 52 prior defined regions of interest (ROIs) that represented six brain networks corresponding to the MTL subsystem (six ROIs), DM subsystem (nine ROIs), Core (nine ROIs), FPCNA subsystem (11 ROIs), FPCNB subsystem (nine ROIs), and DAN (eight ROIs) (see Figure 1). We obtained these ROIs from Matthew L. Dixon. The authors (Dixon et al., 2017, 2018) had used anatomical ROIs created by Yeo and colleagues (Krienen et al., 2014; Yeo et al., 2015) based on their 17-network parcellation derived from the data of 1,000 participants (Yeo et al., 2011) and identified two distinct subsystems within the FPCN based on hierarchical clustering and machine learning classification analyses of within-FPCN FC patterns.

### GCA

**Figure 2:**
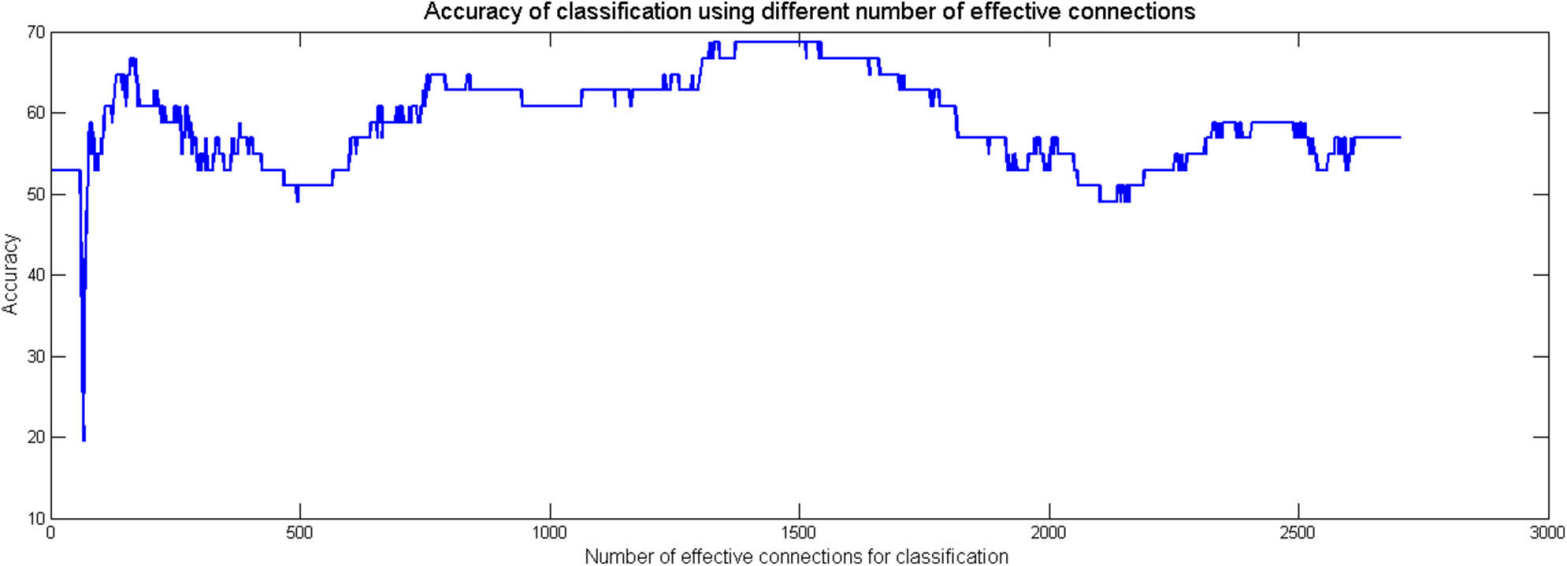
The relationship between classification accuracy and the number of effective connections used in the classification process. The effective connections were ranked according to F scores in descending order.

**Figure 3:**
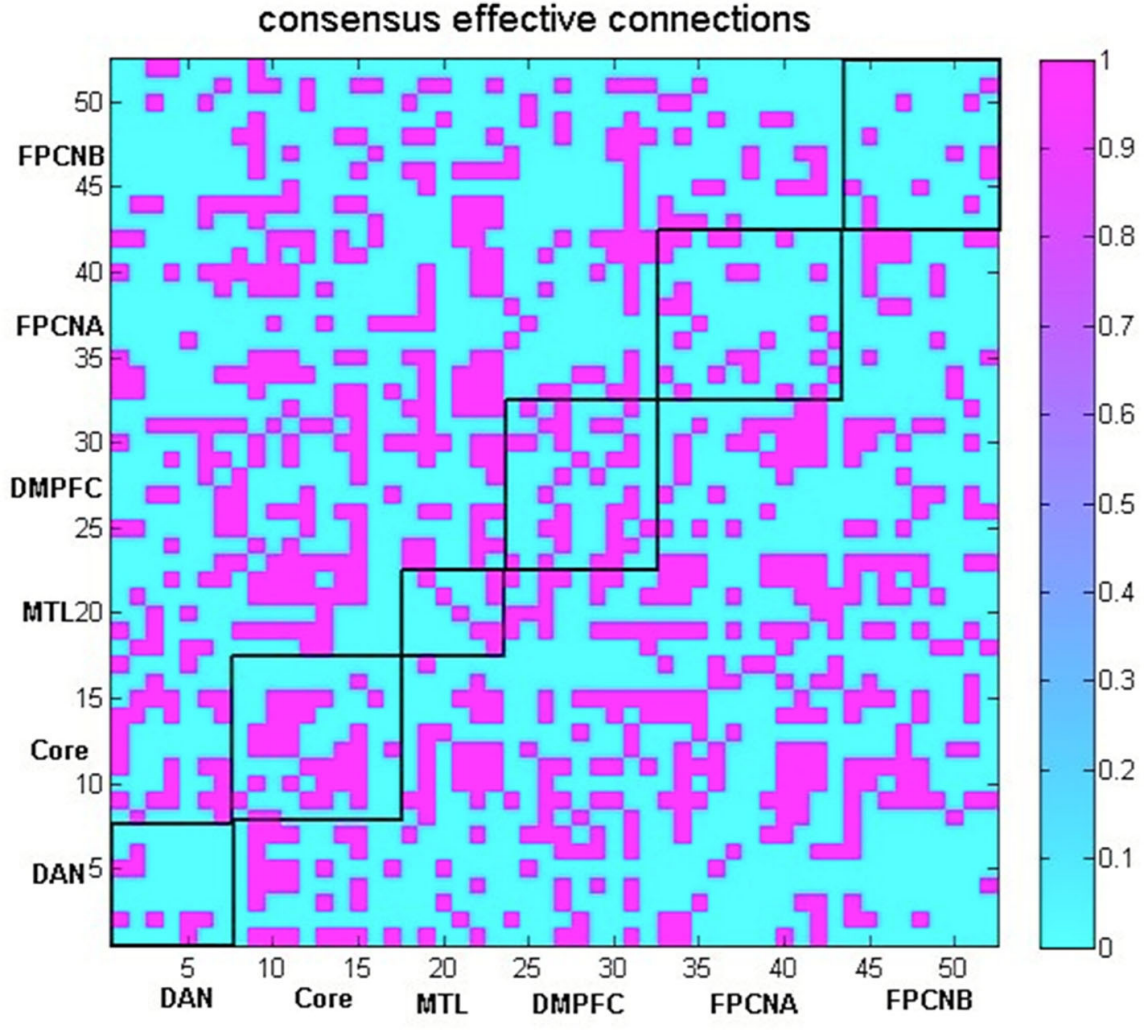
The consensus effective connections within or across the MTL subsystem, DM subsystem, Core, FPCNA subsystem, FPCNB subsystem and DAN (indicated by a 0 or 1).

GCA is an approach used to explore the dynamic causal relationship between two time series (Granger, 1969, 1980). We aimed to examine the bidirectional IFS between any two brain regions in the six networks for the following analyses. The computation was performed using the DynamicBC toolbox (Liao et al., 2014). In this study, we extracted the mean time series from each of these ROIs, and then the time series of all 52 predefined ROIs in the six network subsystems were simultaneously modeled based on the GCA, a bivariate analytic method that characterizes directional functional connections among brain regions to investigate the causal relationship of these regions. To detect reliable Granger causalities between pairs of regions and specifically analyse group differences, a linear SVM classifier was adopted to identify common effective functional connections that stably contributed to the classification between AD patients and healthy controls. These common effective connections were retained for the subsequent analyses. The steps for obtaining these common effective connections were as follows: The effective FC between each pair of regions was used as a classification feature. We performed feature selection by using the F score method for feature ranking (Chen and Lin, 2006; Akay, 2009; Liu et al., 2015). A leave-one-out cross-validation (LOOCV) strategy was used to evaluate the performance of a classifier. For each LOOCV fold, we ranked the features in descending order according to their F scores; 1,320 ranked features were detected, which allowed the linear SVM classifier to reach the highest classification accuracy (see Figure 2). Then, we selected the top 1,320 ranked features in each iteration of the LOOCV for the classification. Since feature ranking was based on a slightly different subset of the data in each iteration of the LOOCV, the final features used in classification differed for each iteration of the LOOCV. We defined common effective connections that were always included in the final feature set in each LOOCV iteration as consensus features. For each participant, 813 consensus features are retained and other connections become 0 for the following network analysis (see Figure 3).

### Regional IFS Analysis

An aim was to examine prominently disrupted brain systems and changes in their interactions with other systems and to explore the underlying cause of these alterations. In the current study, for each ROI, we calculated its inflow/outflow IFS with the other ROIs across all subjects. Similar to the general definition of “in degree” and “out degree,” as provided by previous studies (Sridharan et al., 2008; Stevens et al., 2009), in this study, we extended the definition of regional IFS as follows:

#### Intra-in IFS

Efferent IFS to a region from the other regions in the same network. This causal flow profile identifies regions that are the central targets of the intra-network afferent IF.

#### Intra-out IFS

Afferent IFS from a region to the other regions in the same network. This causal flow profile identifies regions that are the central sources of the intra-network efferent IF.

#### Inter-in IFS

Efferent IFS to a region from all the regions of the other networks. This causal flow profile identifies regions that are the central targets of the inter-network afferent IF.

#### Inter-out IFS

Afferent IFS from a region to all the regions of the other networks. This causal flow profile identifies regions that are the central sources of the inter-network efferent IF. For each ROI, we calculated the sum of intra-in and intra-out as “intra-in+out IFS” and the sum of the inter-in and inter-out as “inter-in+out IFS.” we also calculated the sum of intra-out IFS and inter-out IFS as “intra+inter-out IFS,” as well as the sum of intra-in IFS and inter-in IFS as “intra+inter-in IFS” for every subject. The IFS of these ROIs were compared across groups by using two-sample *t*-tests. We used a statistical significance level of *p* < 0.01. For the ROIs with disrupted intra-in+out IFS, we analyzed the correlation between its intra-in+out IFS and inter-in+out IFS in the patients with AD.

### Inter-Network Effective Connectivity Analyses

To probe the specific changes in the interactions between subsystems. We calculated the forward or feedback IFS of the inter-subsystems for every subject. The inter-subsystem IFS was defined as the total of each inter-regional effective connectivity strength from the ROIs of one network to the ROIs of the other network. The IF between each pair of brain regions is bidirectional; therefore, there are forward and feedback IFS between the two subsystems. To detect group differences in these causal interactions between subsystems, two-sample *t*-tests were performed, and the statistical significance level was set at *p* < 0.01 (the results at *p* < 0.02 were considered statistically significant at *p* < 0.05 after false discovery rate (FDR) correction for multiple comparisons). To further analyse the alterations in the coordination of the causal interactions of the inter-subsystem, we performed correlation analyses on the forward and feedback IFS between subsystems with significant group differences. First, for each group, we used a one-sample *t*-test to verify whether there was a significant correlation between the forward and feedback IFS of the inter-systems. Then, correlation values were compared across groups by using the *Z* score test. In the present study, correlation analyses were performed on the bidirectional IFS between the FPCNA and three DN subsystems.

### Correlation Analyses of Inter-network Dynamic Effective Connectivity

Many researches have found that FPCN as a control network regulated the DN and the DAN and their interactions. In the present study, we were to extend this finding and demonstrate that the IFS initiated from DN subsystems to DAN was dynamically correlated with the causal interactions with the IFS from FPCNA to show the coordination in the inter-system causal interactions and then examined which subsystems play central roles in the alterations of temporal co-evolution of these inter-system causal interactions. Prior work has shown that functionally-relevant FC patterns can be isolated from ~ 60 s of data (Gonzalez-Castillo et al., 2015; Leonardi and Ville, 2015). Based on the inter-system causal interactions with group differences, we selectively examined the IFS values initiated from the Core/DM subsystem to the DAN and from the FPCNA to Core/DM subsystem within 60-s windows, which were then shifted by three timepoints (6 s) each time. Then, within each 60-s window, we averaged the IFS values of these pairs of networks. This provided several time series of inter-system IFS that reflected changes across time in the strength of the causal interactions between these pairs of networks. Furthermore, for every subject, we measured the linear association between the time series of IFS, which was our measure of the co-evolution of these large-scale network subsystem interactions, and these correlation values were converted into *z*-values with the application of Fisher's r-to-z transformation. We used a one-sample *t*-test to examine the significance for each group and compared correlation values across groups to examine whether the temporal co-evolution of sets of inter-subsystem interactions were disrupted in patients with AD. In this study, temporal co-evolution between FPCNA->Core and Core->DAN and between FPCNA->DM and DM->DAN were examined and compared across groups (using two-sample *t*-tests).

### Quantifying the FPCNA Regulatory Effect

To quantify the regulatory effect of the FPCNA on DN subsystems and the interactions between DN subsystems and the DAN, for each subject, we calculated Pearson's correlation between all given ROIs. Then, the partial correlation between pairs of regions within the DN subsystems and between the DN subsystems and DAN was calculated by regressing out the effect of all signals within the FPCNA. Fisher's z transform was also applied to both the full correlation and partial correlation values. The strength of the intra-subsystem FC was defined as the total inter-regional FC strength within the selected subsystem, while the FC strength of the inter-subsystem was defined as the total FC strength between any two ROIs of the two selected subsystems. For the NC group, we compared the full with the partial correlation strength within the DN subsystems and between the DN subsystems and DAN using paired *t*-tests to clarify the regulatory role of the FPCNA. The differences between the full and the partial correlation strength of the intra- and inter-subsystems were then taken as indicators of the regulatory effects of the FPCNA on the DN subsystems and interactions between the DN subsystems and DAN (Gao and Lin, 2012), which was compared across groups (using two-sample *t*-tests) to detect the change in the regulatory effect of the FPCNA in patients with AD.

### SVM Classification Analysis

To explore which systems contribute the most to the discrimination between patients with AD and healthy elderly, a linear kernel SVM classifier was adopted for classification to reduce the risk of overfitting the data (Pereira et al., 2009). The SVM classifier was implemented using the LIBSVM toolbox (Chang and Lin, 2011), with a default value for the parameter C (i.e., *C* = 1). The calculated indicators of the brain networks that had higher absolute t-statistics were selected as the classification features in the classifier. Due to our limited number of samples, an LOOCV strategy was used to estimate the classification accuracy. In each iteration of the LOOCV, an example from total samples was used as the testing set, while the remaining examples were used as the training set. Additionally, to assess whether the computed classification accuracy is statistically significant, the statistical significance of the classification results is evaluated using permutation test (Golland and Fischl, 2003). In the current analysis, the class labels (i.e., AD vs. NC) of the training data were permuted 1,000 times at random prior to training and then the same entire classification process was carried out with each set of permuted class labels. If *p* < 0.05, the result was thought to be significant. In this study the *p* = 0 after using permutation test.

## Results

### Group Differences in the Regional IFS

An aim was to better evaluate the prominently disrupted brain networks and changes in their interactions with other systems. For every ROI, we calculated its “intra-in/out,” “intra-in+out,” “inter-in/out,” “inter-in+out,” “intra+inter-out,” and “intra+inter-in” IFS, and the number of ROIs of the large-scale brain networks showing significant group differences in these indicators are shown in Table 2. Due to too many significant results, in the present research, we selectively presented only ROIs and their statistics (i.e., only a few ROIs (*n* < 3) with group differences in a certain indicator or *p* ~= 10^−4^). ROIs within the DN showed decreased regional IFS in the patients with AD compared with the NCs: decreased intra-in IFS was found in the PCC_L [*t*_(49)_ = −4.12, *p* = 1.4^*^10^−4^], vPCC_L [*t*_(49)_ = −4.20, *p* = 1.2^*^10^−4^], vPCC_R [t_(49)_ = −3.74, *p* = 4.7^*^10^−4^], etc. Weaker intra-out IFS was observed in the PCC_L [*t*_(49)_ = −3.57, *p* = 8.0^*^10^−4^], PCC_R [*t*_(49)_ = −4.00, *p* = 2.5^*^10^−4^], vPCC_L [*t*_(49)_ = −3.58, *p* = 7.9^*^10^−4^], vPCC_R [*t*_(49)_ = −3.53, *p* = 9.1^*^10^−4^], pIPL_R (t(49) = −3.55, p = 8.6^*^10^−4^), DM_L [*t*_(49)_ = −3.80, *p* = 4.0^*^10^−4^], etc. Reduced intra-in+out IFS was found in the PCC_L [*t*_(49)_ = −3.91, *p* = 2.8^*^10^−4^], PCC_R [*t*_(49)_ = −3.78, *p* = 4.2^*^10^−4^], VPCC_R [*t*_(49)_ = −3.63, *p* = 6.7^*^10^−4^], pIPL_R [*t*_(49)_ = −3.78, *p* = 4.1^*^10^−4^], etc. Decreased inter-in IFS, inter-out IFS and inter-in+out IFS were also found in ROIs within the DN. Additionally, the PCC_L [*t*_(49)_ = −3.68, p = 5.8^*^10^−4^], PCC_R [*t*_(49)_ = −4.16, *p* = 1.3^*^10^−4^], DM_L [*t*_(49)_ = −3.78, *p* = 4.3^*^10^−4^], etc., within the DN showed decreased intra+inter-out IFS; the PCC_L [*t*_(49)_ = −3.76, *p* = 4.6^*^10^−4^], PCC_R [*t*_(49)_ = −3.92, *p* = 2.7^*^10^−4^], vPCC_L [*t*_(49)_ = −3.93, *p* = 2.6^*^10^−4^], etc., within the DN exhibited reduced intra+inter-in IFS in the patients with AD. ROIs in the FPCNA showed decreased inter-in IFS, inter-out IFS and inter-in+out IFS, whereas decreased intra-out IFS was found only in the aIPL_L [*t*_(49)_ = −3.01, *p* = 0.0041] in the patients with AD relative to NCs, and no ROIs of the FPCNA showed group differences in intra-in and intra-in+out IFS. Regions of the FPCNA also showed decreased intra+inter-in and intra+inter-out IFS in the patients with AD. Decreased inter-in IFS and inter-in+out IFS occurred in ROIs of the FPCNB; however, no group differences were observed in inter-out IFS. Only the Right inferior frontal sulcus [*t*_(49)_ = −2.84, *p* = 0.0065, *t*_(49)_ = −3.11, *p* = 0.0032] showed decreased intra-in and intra-out IFS; moreover, decreased intra-in+out IFS was observed in the Right inferior frontal sulcus [*t*_(49)_ = −3.13, *p* = 0.0029] and aIFS_L [*t*_(49)_ = −2.78, *p* = 0.0077]. Regions in the FPCNB also showed decreased intra+inter-in IFS, and decreased intra+inter-out IFS was found only in the aIFS_L [*t*_(49)_ = −3.14, *p* = 0.0029] and Right inferior frontal sulcus [*t*_(49)_ = −2.95, *p* = 0.0049] in the patients with AD.

**Table 2:** The number of ROIs in the brain networks with significant group differences in regional IFS (*p* < 0.01).

**Regional IFS**	**DN**	**FPCNA subsystem**	**FPCNB subsystem**	**DAN**
Intra-in IFS	14	0	1	0
Intra-out IFS	14	1	1	1
Intra-in+out IFS	14	0	2	0
Inter-in IFS	6	4	4	1
Inter-out IFS	6	3	0	1
Inter-in+out IFS	6	4	4	2
Intra+inter-in IFS	12	4	4	1
Intra+inter-out IFS	14	5	2	0

**Figure 4:**
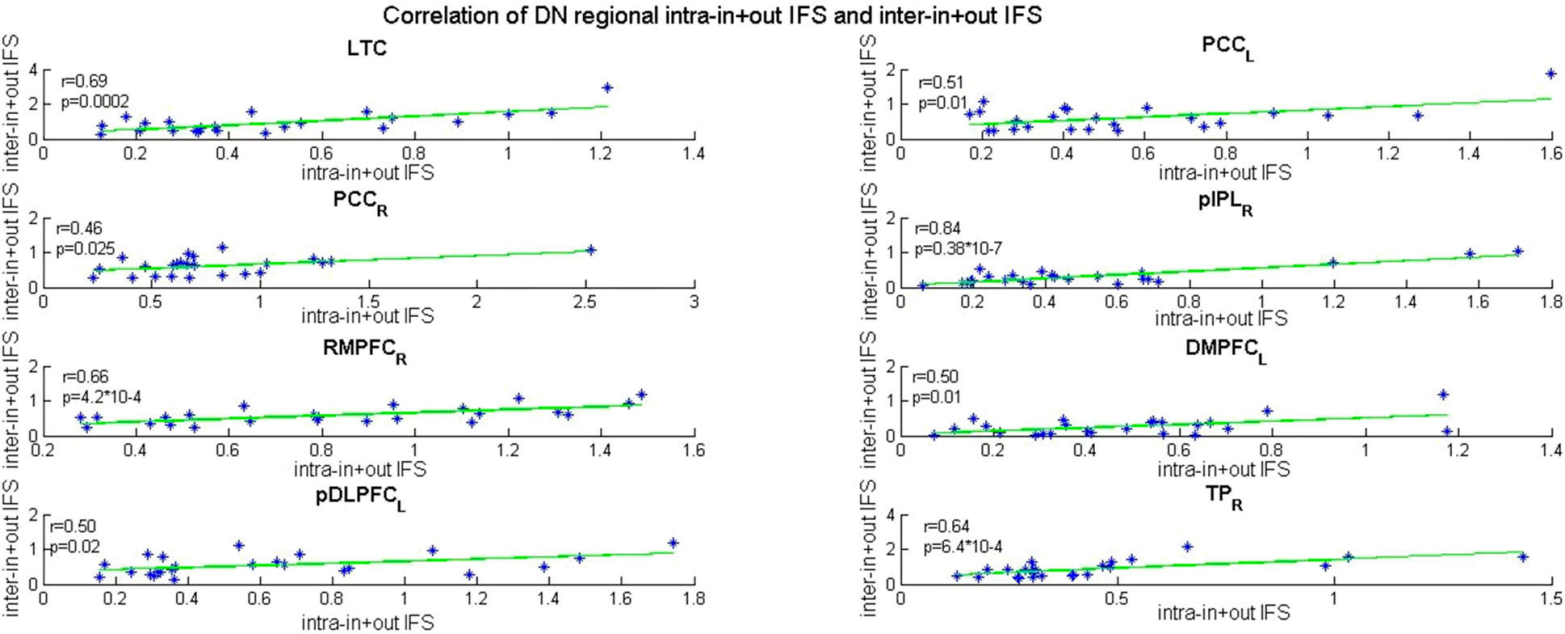
Correlation of decreased DN regional intra-in+out IFS and inter-in+out IFS in AD subjects (*P* < 0.05, uncorrected for multiple comparisons). LTC, lateral temporal cortex; PCC, posterior cingulate cortex; pIPL, posterior inferior parietal lobule; RMPFC, rostromedial prefrontal cortex; DM, dorsomedial prefrontal cortex; pDLPFC, posterior dorsolateral prefrontal cortex; TP, temporopolar cortex; L, left hemisphere; R, right hemisphere; r, Pearson correlation coefficient.

The aMT_L in the DAN showed decreased inter-in IFS [*t*_(49)_ = −2.82, *p* = 0.0070] and inter-out IFS [*t*_(49)_ = −2.91, *p* = 0.0054), and the aMT_L [*t*_(49)_ = −3.00, *p* = 0.0043) and PrCv_R [*t*_(49)_ = −2.80, *p* = 0.0071] showed decreased inter-in+out IFS. Increased intra-out IFS was found only in the aMT_R [*t*_(49)_ = 2.78, *p* = 0.0076], and no group difference was observed in the intra-in IFS and intra-in+out IFS. Decreased intra+inter-in IFS was found in the PrCv_R [*t*_(49)_ = −2.98, *p* = 0.0045], whereas no group difference was observed in intra+inter-out IFS in the patients with AD. To explore whether the disrupted intra-DN IFS was related to its inter-system interaction, for the ROIs of the DN with significant group differences in intra-in+out IFS, we performed correlation analyses between the intra-in+out IFS and inter-in+out IFS in the AD group. We found that the intra-in+out IFS of eight regions in the DN was significantly associated with its inter-in+out IFS (see Figure 4) including the LTC (*r* = 0.69, *p* = 2.0^*^10^−4^), PCC_L (*r* = 0.51, *p* = 0.01), PCC_R (*r* = 0.46, *p* = 0.025), pIPL_R (*r* = 0.84, *p* = 0.38^*^10^−7^), RMPFC_R (*r* = 0.66, *p* = 4.2^*^10^−4^), DM_L (*r* = 0.50, *p* = 0.01), pDLPFC_L (*r* = 0.50, *p* = 0.02), and TP_R (*r* = 0.64, *p* = 6.4^*^10^−4^).

### Group Differences in the Causal Interactions of Inter-systems

**Figure 5:**
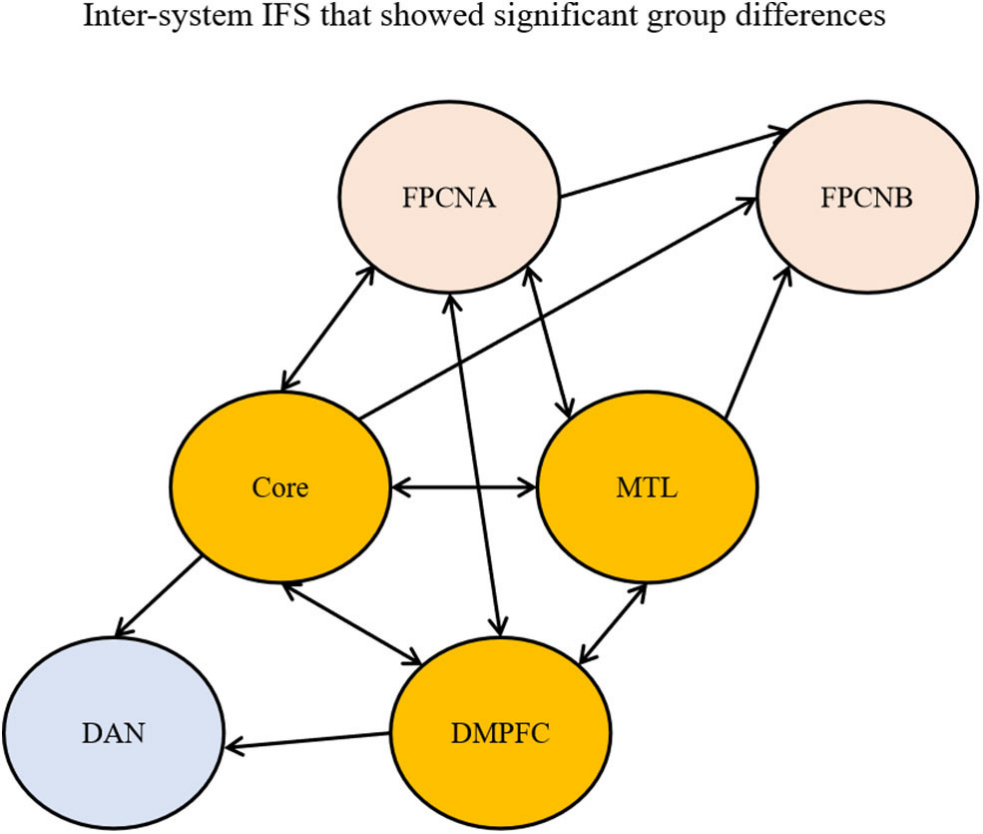
Inter-subsystem IFS showing significant group differences (*p* < 0.01). A single arrow indicates that the unidirectional connection between networks was decreased in AD. Double arrows indicate that the reciprocal connections between networks were decreased in AD. FPCNA, frontoparietal control network A; FPCNB, frontoparietal control network B; DM, dorsomedial prefrontal cortex; MTL, medial temporal lobe subsystem; DAN, dorsal attention network.

The specific changes in causal interactions among these networks were explored. The inter-system IFS values with significant group differences are shown in Figure 5. Compared with the healthy elderly, the AD patients showed decreased bidirectional IFS among the DN subsystems, including the IFS between the Core and MTL subsystem [Core->MTL: *t*_(49)_ = −3.85, *p* = 3.48^*^10^−4^; MTL->Core: *t*_(49)_ = −4.14, *p* = 1.36^*^10^−4^], between the Core and DM subsystem [Core->DM: *t*_(49)_ = −2.88, *p* = 0.0059; DM->Core: *t*_(49)_ = −2.92, *p* = 0.0052], and between the MTL and DM subsystem [MTL->DM: *t*_(49)_ = −2.89, *p* = 0.0058; DM->MTL: *t*_(49)_ = −3.00, *p* = 0.0043] in the AD patients. We also found both decreased forward and feedback IFS between the Core and FPCNA [Core->FPCNA: *t*_(49)_ = −2.69, *p* = 0.0097; FPCNA->Core: *t*_(49)_ = −2.78, *p* = 0.0076], between the MTL subsystem and FPCNA [MTL->FPCNA: *t*_(49)_ = −3.08, *p* = 0.0034; FPCNA->MTL: *t*_(49)_ = −3.14, *p* = 0.0028], and between the DM subsystem and FPCNA [DM->FPCNA: *t*_(49)_ = −2.87, *p* = 0.0060; FPCNA->DM: *t*_(49)_ = −2.74, *p* = 0.0086) in the AD patients relative to the healthy elderly. Additionally, the results revealed that the IF initiated from both the Core [*t*_(49)_ = −2.95, *p* = 0.0049] and DM [*t*_(49)_ = −3.36, *p* = 0.0015) subsystem to the DAN were decreased; the IF initiated from the Core [*t*_(49)_ = −2.99, *p* = 0.0044], MTL subsystem [*t*_(49)_ = −2.95, *p* = 0.0049] and FPCNA subsystem [*t*_(49)_ = −2.74, *p* = 0.0085] to the FPCNB subsystem were also decreased in the AD patients compared with the healthy elderly.

**Figure 6:**
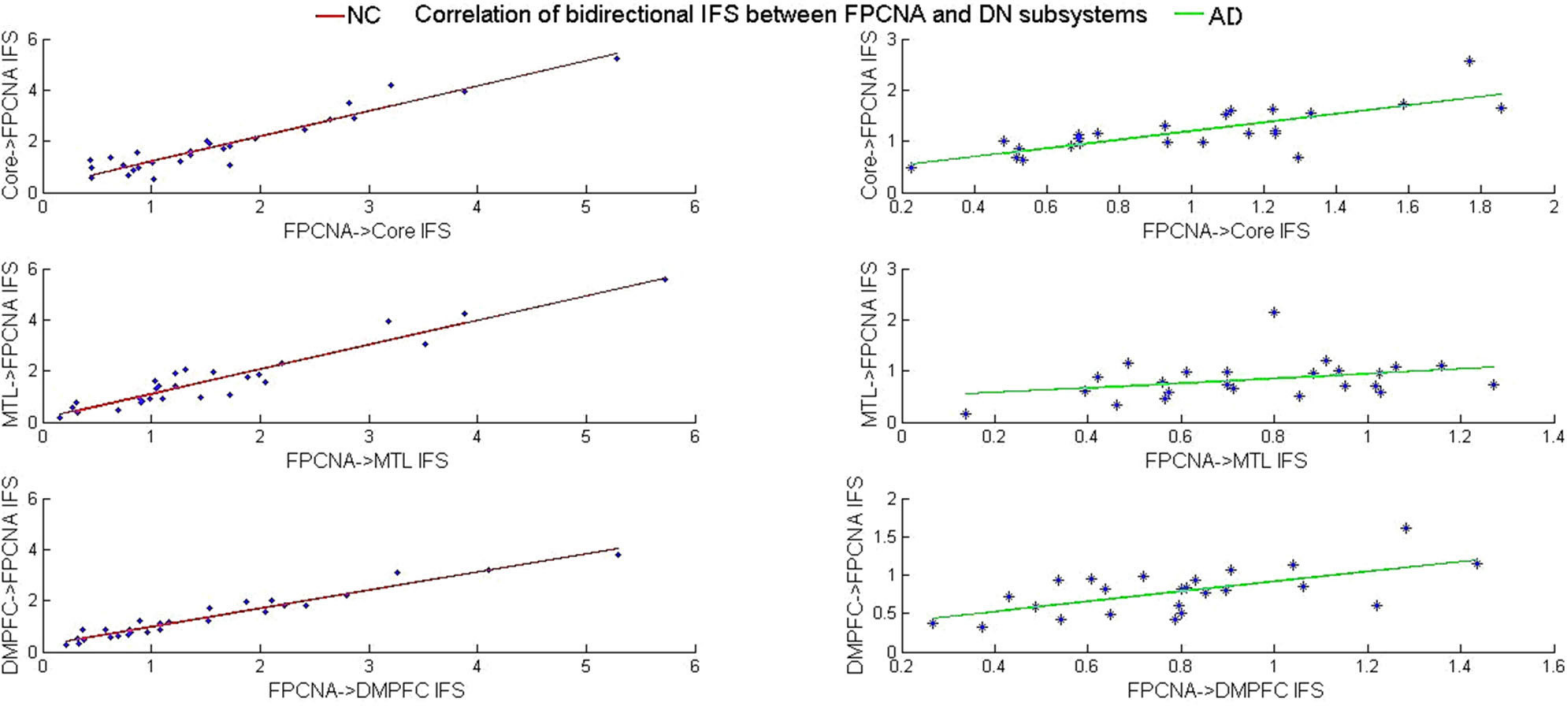
The correlation of forward and feedback IFS between the FPCNA and three DN subsystems in the NC and AD groups.

To explore the alterations in the coordination of the disrupted causal interactions of particular pairs of networks, the correlations of the forward and feedback IFS between the FPCNA and DN subsystems were calculated and then compared across groups. We found that forward and feedback IFS between the FPCNA subsystem and three DN subsystems [DM subsystem: (*r* = 0.97, *p* = 0); Core: (*r* = 0.95, *p* = 3.6 ^*^10^−14^); and MTL subsystem: (*r* = 0.95, *p* = 2.1^*^10^−14^)] were significantly correlated in healthy elderly group (see Figure 6). Finally, the results revealed decreased correlation values of the bidirectional IFS between the FPCNA and the MTL subsystem (*Z* = −5.05, *p* = 4.40^*^10^−7^), DM subsystem (*Z* = −4.66, *p* = 3.10^*^10^−6^), and Core (*Z* = −2.61, *p* = 0.0090) in the AD patients relative to the healthy elderly (see Figure 6).

### Group Differences in Temporal Co-evolution of the Inter-system Causal Interactions

**Figure 7:**
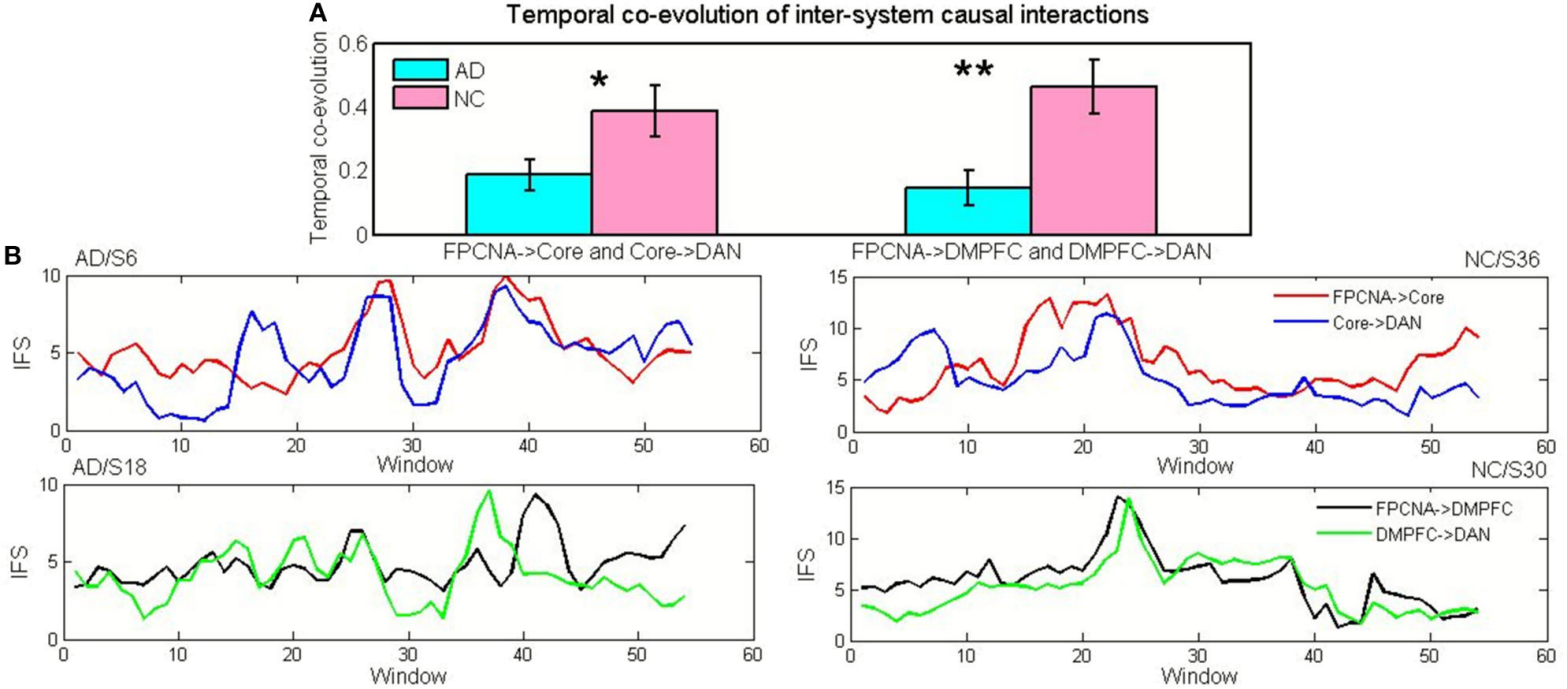
Temporal co-evolution of inter-system causal interactions in the NC and AD groups. **(A)** Temporal co-evolution between FPCNA->Core IFS and Core->DAN IFS and between FPCNA->DM IFS and DM->DAN IFS in the NC and AD groups (*p < 0.05, **p < 0.005). Error bars represent the standard error of the mean. **(B)** Data for four randomly chosen example participants demonstrating IFS from FPCNA to Core and IFS from Core to DAN (Top) as well as IFS from FPCNA to DM and IFS from DM to DAN (Bottom) within successive 60-second windows in the AD and NC groups.

We selectively assessed correlations between the dynamic effective connections of inter-subsystem to measure the temporal co-evolution of sets of inter-subsystem causal interactions, which was then compared across groups. The results showed that IFS initiated from the FPCNA to the Core was significantly correlated with the IFS initiated from the Core to the DAN [r_mean_ = 0.39, *p* = 3.5^*^10^−6^, *t*_(26)_ = 5.1], and a significant dynamic correlation [r_mean_ = 0.46, *p* = 2.7^*^10^−5^, *t*_(26)_ = 5.9] was also found between the FPCNA->DM subsystem IFS and the DM subsystem->DAN IFS in the healthy elderly group. Moreover, temporal co-evolution between FPCNA->Core IFS and Core->DAN IFS [*t*_(49)_ = −2.2, *p* = 0.04), as well as between FPCNA->DM IFS and DM->DAN IFS [*t*_(49)_ = −3.2, *p* = 0.0025), was significantly decreased in the AD patients compared with the healthy elderly (see Figure 7).

### Group Differences in the Regulatory Effect of the FPCNA

**Figure 8:**
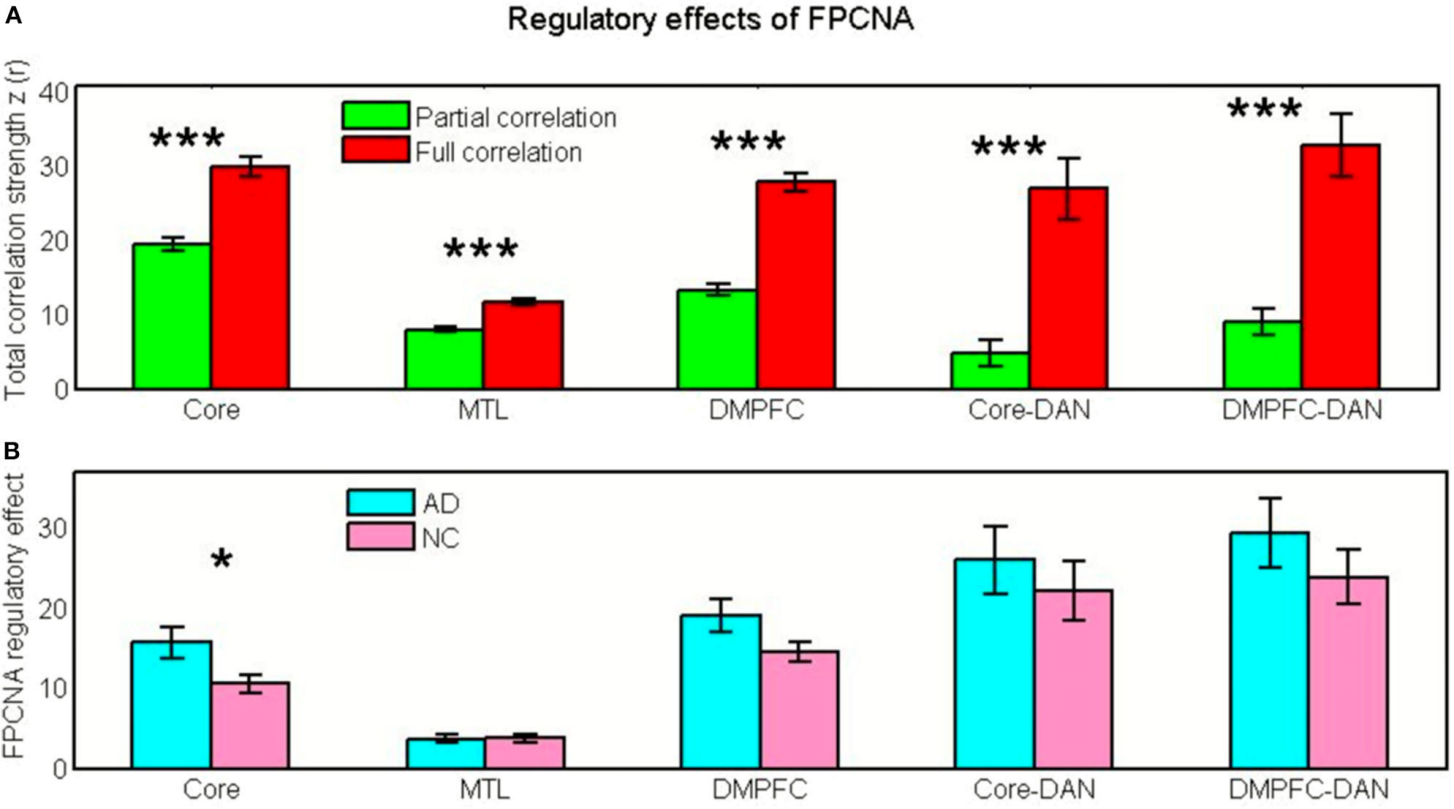
The regulatory effects of the FPCNA. **(A)** In the NC group, full and partial correlation strength within the Core, MTL subsystem, DM subsystem, between the Core and DAN, and between the DM subsystem and DAN. **(B)** The regulatory effect of the FPCNA on the Core, MTL subsystem, DM subsystem, interactions between the Core and DAN, and between the DM subsystem and DAN in the NC and AD groups (*p < 0.05, **p < 0.005, ***p < 0.001).

The changes in the regulatory effect of the FPCNA were examined in the AD patients relative to the healthy elderly. Compared with the full correlation, the FC strength within the Core [*t*_(26)_ = −8.80, *p* = 2.8^*^10^−9^], MTL subsystem [*t*_(26)_ = −7.66, *p* = 4.0^*^10^−8^], and DM subsystem [*t*_(26)_ = −11.11, *p* = 2.3^*^10^−11^], between the DM subsystem and DAN [*t*_(26)_ = −6.94, *p* = 2.3^*^10^−7^], and between the Core and DAN [*t*_(26)_ = −6.08, *p* = 2.0^*^10^−6^] after removing the role of the FPCNA were significantly decreased in healthy elderly group (see Figure 8A). In the AD patients, the FPCNA showed a higher regulatory effect on the Core [*t*_(49)_ = 2.31, *p* = 0.025] than in the healthy controls, whereas no group differences were found in the regulating effect of the FPCNA on the MTL subsystem [*t*_(49)_ = −0.053, *p* = 0.96], DM subsystem [*t*_(49)_ = 1.89, *p* = 0.064], interaction between the Core and DAN [*t*_(49)_ = 0.70, *p* = 0.49], or interaction between the DM and DAN [*t*_(49)_ = 1.01, *p* = 0.32] (see Figure 8B).

### Network Roles in the Classification Between AD Patients and NCs

**Figure 9:**
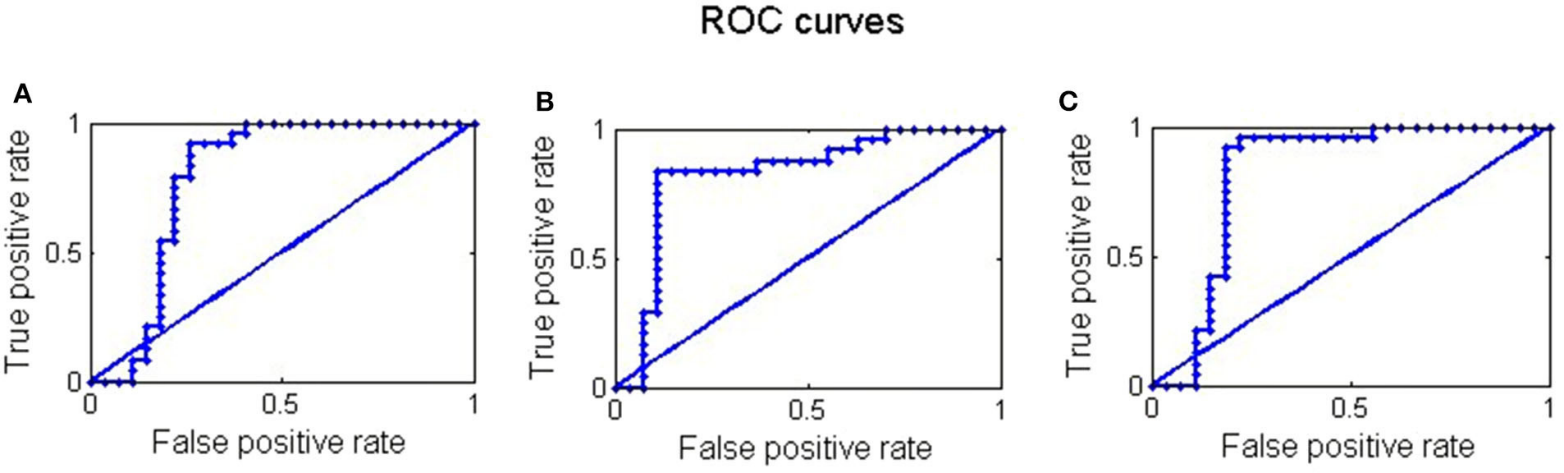
ROC curves of the linear SVM classifier using **(A)** intra-in IFS of the vPCC_L, **(B)** intra-in+out IFS of the PCC_L, and **(C)** intra+inter-out IFS of the PCC_R within the DN as classification features. PCC, posterior cingulate cortex; vPCC, ventral posterior cingulate cortex.

The network that made the strongest contribution to the classification between the two groups was identified. The SVM classifier was adopted for classification using indicators with higher absolute t-statistics as the features. As shown in Table 3 and Figure 9, the linear SVM classifier achieved an accuracy of 82%, with a sensitivity of 91%, a specificity of 74%, and an area under the receiver operating characteristic (ROC) curve value of 79% by using the intra-in IFS of the vPCC_L within the DN as the classification feature. When we used intra-in+out IFS of the PCC_L within the DN as the feature, the SVM classifier reached an accuracy of 74%, a sensitivity of 83%, a specificity of 89%, and an area under the curve (AUC) value of 82%. The linear SVM classifier attained an accuracy of 76%, with a sensitivity of 92%, a specificity of 81%, and an AUC value of 82 when we used the intra+inter-out IFS of the PCC_R within the DN as the features.

**Table 3:** Classification performance using linear SVM classifiers with the regional IFS of ROIs within DN as classification features.

**Classification feature**	**Accuracy**	**Sensitivity**	**Specificity**	**AUC**
Intra-in IFS of the vPCC_L	82%	91%	74%	79%
Intra-in+out IFS of the PCC_L	74%	83%	89%	82%
Intra+Inter-out IFS of the PCC_R	76%	92%	81%	82%

## Discussion

The current study suggests that prominently disrupted DN subsystems initiated their disordered interactions with FPCN subsystems and DAN and caused the FPCNA to have a higher regulatory burden. First, the regional IFS analysis revealed that the regional IFS of most ROIs within the DN significantly decreased in the AD patients, and the disrupted intra-DN regional IFS was closely related to inter-system regional IFS. Additionally, some ROIs in the FPCN subsystems and DAN also showed decreased inter-system regional IFS, but only a few showed decreased intra-system regional IFS. Moreover, the inter-system effective connectivity analysis demonstrated that the DN subsystems showed extensive decreased effective connections with the FPCN subsystems and DAN in a different pattern and that correlation values of the forward and feedback IFS between the FPCNA and DN subsystems significantly decreased in the AD patients compared with the healthy elderly. Furthermore, DN subsystems played a central role in the changes in the temporal co-evolution of sets of inter-system interactions in AD; the FPCNA exhibited a higher regulatory effect on the Core in the AD patients; and the DN made the strongest contribution to the classification between the AD and NC groups.

### Prominently Impaired Intra-DN Regional IFS Is Associated With Inter-system Regional IFS in Patients With AD

The regional IFS analysis results suggested that the DN was prominently disrupted in the AD patients, and other systems outside the DN also had a certain degree of disruption. Consistent with our findings, previous studies revealed mainly impaired FC and activity abnormalities as well as structural atrophy of the DN (Greicius et al., 2004; Buckner et al., 2005, 2009; Wang et al., 2006; Zhou et al., 2008; Yu et al., 2017). Some studies have proposed that the DN was the first large-scale system shown to be disrupted in AD (Greicius et al., 2004; Buckner et al., 2005; Rombouts et al., 2005; Sorg et al., 2007; Sheline et al., 2010; Zhang et al., 2010), and other networks were involved in succession (Agosta et al., 2011; Thomas et al., 2014; Jones et al., 2015; Wang et al., 2015). Our results also showed that disrupted intra-system regional IFS of ROIs in the DN were associated with the inter-system regional IFS. A few studies have directly explored the relationships between intra- and inter-system functional connections; however, Avelar-Pereira et al. (2017) found that the degree of DN-DAN anti-correlation during a task was associated with DN neural activity during rest. Additional analyses have proposed that disruptions in the DN initiated disordered FC with other networks that were initially unaffected by AD (Brier et al., 2012, 2014). Our findings support the hypothesis that the prominent disruption of the DN affected its inter-system causal interactions.

### Disrupted Causal Interactions of Inter-systems in Patients With AD

The inter-system effective connectivity analysis showed that the bidirectional IFS values among the DN subsystems and between the three DN subsystems and FPCNA were decreased in the AD patients, additionally, effective connections initiated from the Core, MTL subsystem and FPCNA to the FPCNB subsystem, from the Core and DM subsystem to DAN were decreased. These results suggested that the DN subsystems play a crucial role in the alterations of inter-subsystem interactions. Consistent with our findings, previous researchers have reported that decreased effective connectivity was found between the DN and other systems in patients with AD (Liu et al., 2012). Resting-state FC analysis also revealed abnormal connectivity between the DN and DAN and FPCN in AD patients (Brier et al., 2012; Wang et al., 2015; Zhu et al., 2016). Recent studies have proposed that the DN couples with the FPCN to generate and sustain an internal train of thought (Smallwood et al., 2012). Dixon et al. (2018) indicated that the FPCNA subsystem was preferentially coupled with the DN to regulate internal thoughts and emotions such as abstract thinking (Christoff et al., 2009; Fox et al., 2015), relational reasoning (Christoff et al., 2001; Vendetti and Bunge, 2014), mentalizing (Bhatt et al., 2010), episodic memory (Fornito et al., 2012), and future planning (Spreng and Grady, 2010). The disruption of directional IFS between the FPCNA and DN subsystems may result in cognitive impairment of these introspective processes. Studies have suggested that the core of the DN shares functional properties with both the MTL and DM subsystems involved in self-referential processing, emotion evaluation, and social and mnemonic processes (Andrews-Hanna et al., 2014). In this study, in addition to decreased effective connectivity with the FPCNA subsystem, the Core also exhibited extensive disrupted effective connections with the DAN and FPCNB subsystems. In line with previous findings, Zhang et al. (2010) suggested that impairments in PCC (one of the critical regions of the Core) FC changes with the progression of AD. Previous studies have found that the anti-correlation between the DN and the DAN is attenuated in AD patients (Zhou et al., 2010; Agosta et al., 2011; Brier et al., 2012; Thomas et al., 2014; Zhu et al., 2016). Intrinsically, the anti-correlated DAN and DN inversely subserve externally and internally directed cognition, respectively (Spreng et al., 2010; Andrews-Hanna, 2012), and this anti-correlation pattern may play an important role in flexibly allocating attentional resources. Dixon et al. (2017) revealed that the DAN showed weak negative FC with the Core but was uncorrelated with the DM and MTL subsystems. Hence, the disrupted effective connectivity from the Core to the DAN in AD patients might lead to impaired function at the clinical level, which may partly explain the attention deficits observed with AD patients. Additionally, decreased IFS was also found from the DM subsystem to the DAN and from the Core and MTL subsystems to the FPCNB subsystem. Our results also indicated that distinct DN subsystems have different patterns of disrupted inter-system interactions, which further proved the heterogeneity of these large-scale networks' functional-anatomic organization (Buckner et al., 2008; Uddin et al., 2009; Sestieri et al., 2011; Yeo et al., 2011; Andrews-Hanna, 2012; Kim, 2012; Salomon et al., 2014; Dixon et al., 2018), and our findings regarding the changes in these large-scale network subsystem interactions may provide a greater understanding of the system-level pathogenesis of AD. The DM subsystem and DAN are involved in mentalizing and perceptual processing, respectively, sometimes operate in concert, making inferences about others' thoughts by analyzing the perceptual input of body language, facial expression, and eye gaze (Baron-Cohen et al., 2001; Dixon et al., 2017). The disruption of effective connections between the DM subsystem and the DAN may affect AD patients' normal cognitive abilities. The FPCNB is preferentially coupled with the DAN to engage in regulating visuospatial perception and action during physical interactions with the environment; however, it was also shown to be aligned with the DN in some contexts (Stokes et al., 2013; Dixon et al., 2018). The decreased IFS from the MTL and Core to the FPCNB during rest can also affect its IFS during a task; therefore, we speculated that the disruptions possibly led to limited task-related flexibility of inter-system interactions. In this study, we also found decreased IFS from the FPCNA to the FPCNB subsystem. In particular, the FPCNB has been more related to complex tasks, and the FPCNA may recruit the FPCNB when performing highly complex, perceptually focused tasks (Dixon et al., 2018). Disruption of effective connectivity from the FPCNA to the FPCNB subsystem may, to some degree, explain AD patients' impaired ability to complete complex cognitive and executive function tasks. To further explore the changes in coordination in these disrupted causal interactions of inter-systems, we compared correlation values of forward and feedback IFS between the FPCNA and DN subsystems and found that these correlation values were significantly reduced in the AD patients. Previous studies scarcely examine changes in the coordination of causal interactions in inter-systems. Similar to self-organizing and homeostatic feedback control in other biological systems, such as body temperature control and the body's immune system, we assumed that the control system of the FPCNA showed feedback regulation of the DN subsystems (Brun et al., 2009; Cole et al., 2014). Our results clarified a significant correlation of bidirectional IFS between the FPCNA and DN subsystems and the meditation role of the FPCNA on the DN subsystems (the latter will be elaborated on in detail below) in the healthy elderly group, which further supports this hypothesis. Therefore, in view of our findings, we speculated that the disrupted DN subsystems had difficulty effectively responding to signals from the FPCNA meditation and could not correctly convey the feedback information to the FPCNA in AD patients. For example, in some contexts, the disrupted DN cannot correctly transmit the inhibitory feedback signal to the FPCNA, and the DN would fail to be deactivated; meanwhile, to maintain the normal function of the DN, the FPCNA continues to constantly attempt to regulate the damaged DN subsystems (Spreng and Schacter, 2012; Cole et al., 2014). Our findings suggested that the DN subsystems not only initiated disrupted inter-system causal interactions but also caused incoordination in the causal interactions between the DN subsystems and the FPCNA in AD patients.

### Disordered Temporal Co-evolution of Sets of Inter-system Causal Interactions

Abundant evidence has suggested that the FPCN may serve as a “switch” that actively engages or disengages the DN and DAN to regulate the balance between them (Spreng et al., 2010, 2013; Gao and Lin, 2012; Cole et al., 2013b; Shaw et al., 2015). In the present study, we also extended this finding and demonstrated that variation across time in the IFS initiated from the FPCNA subsystem to Core was related to that from the Core to DAN and that variation across time in the IFS initiated from the FPCNA subsystem to the DM subsystem was associated with that from the DM subsystem to DAN in the healthy elderly group. The DM subsystem and Core are involved in processing high-level conceptual information associated with the self or others (Binder et al., 2009; D'Argembeau et al., 2010, 2012; Denny et al., 2012; Andrews-Hanna et al., 2014; Simony et al., 2016). The FPCNA IFS patterns being tightly coupled with the IFS from the Core/DM subsystem to DAN changes across time could potentially reflect moment-to-moment shifts in the distribution of attention between perceptual information and internally oriented conceptual thought (Dixon et al., 2017). In the present study, we found that temporal co-evolution between FPCNA->Core IFS and Core->DAN IFS as well as between FPCNA->DM IFS and DM->DAN IFS was significantly decreased in the AD patients. The disordered temporal co-evolution of these large-scale network subsystem interactions in the AD group may reflect impaired flexibility and reconfiguration abilities of the interactivity between RSNs, which may lead to deficits in cognitive and executive function (Cocchi et al., 2013; Dwyer et al., 2014). The results also suggested that DN subsystems play central roles in the disordered temporal co-evolution of these inter-system causal interactions. Therefore, our findings further demonstrated that significantly disrupted DN initiated disordered causal interactions of the inter-system.

### Higher Regulatory Burden of the FPCN on the Core in Patients With AD Relative to NCs

Extensive evidence has identified the FPCN as a control system showing extensive functional connectivity to regulate other systems (Cole et al., 2010, 2013a,b; Power et al., 2011). This study suggested a regulatory ability of the FPCNA on DN subsystems and interactivity between the DM subsystem and DAN, as well as an interaction between the Core and DAN in the healthy elderly group. We assumed that the constant regulation of the FPCNA on the disrupted DN subsystems and interactions between systems possibly leads itself to a higher regulatory burden in AD patients. To test this hypothesis, we compared its meditating effect across the groups. We found that the FPCNA showed a higher regulatory effect on the Core, but the regulatory effects of the FPCNA on other DN subsystems and interactions between systems showed no group differences. This finding is consistent with previous observations that enhanced connectivity occurred mainly between the PCC and the prefrontal-parietal cortex in AD patients (Zhang et al., 2010; Franzmeier et al., 2017). Studies have reported that increased FC between the FPCN and DN is coupled with reduced connectivity within the DN to maintain better memory performance (Grady et al., 2016). The FPCN has recently been proposed to be related to higher reserve capacity (Franzmeier et al., 2017). The increased regulating effect of the FPCNA may compensate for the deficit in the function of the DN. Therefore, the structural and functional integrity of the FPCNA plays a crucial role in maintaining normal cognitive function (Cole et al., 2014). In the present study, a few ROIs in the FPCN subsystems were also impaired in the AD patients. We speculated that the continuous high-load regulation of damaged systems may cause damage to the regulatory system and that this disruption of the FPCN will lead to more severe cognitive deficits in AD patients. Therefore, the higher regulatory burden of the FPCN subsystem may serve as a warning that the recently influential treatment based on transcranial magnetic stimulation (TMS) that stimulates regions of the FPCN to activate the downstream regions of DN (Chen et al., 2013) may help to improve patients' cognitive function in a short time and will possibly have the risk of irreversibly exacerbating the AD. Our findings not only support the hypothesis that the significantly disrupted DN subsystem causes a higher regulatory burden on the FPCNA and but also possibly provide a valid warning about the approach to treat AD by stimulating brain regions within the FPCN via TMS.

### The Core and MTL Subsystem Made the Strongest Contributions to the Classification Between AD Patients and NCs

To identify which systems made the most contribution to the classification between the AD and the healthy elderly groups, we applied an SVM linear classifier to differences with the higher absolute t-statistics. The results showed that the regional IFS of the regions within the Core (PCC_L, PCC_R) and MTL subsystems (vPCC) had higher sensitivity and specificity for classification than each of the other examined features. Our findings further demonstrated that the brain regions within the MTL subsystem and Core may be more susceptible to AD. The present results were supported by our previous findings that the MTL subsystem was significantly disrupted and that the PCC plays an important role in the disruption of FCs in AD (Qi et al., 2018). Seminal studies have reported that the external attentional state is related to the PCC; in contrast, the vPCC supports various aspects of internally oriented cognition, including the retrieval of episodic and semantic memories (Hampson et al., 2006; Gilbert et al., 2007; Hahn et al., 2007; Leech and Sharp, 2014). The interactions between the PCC and vPCC and other systems are the key to regulating the balance between internal and external attentional focus (Leech et al., 2011). The disrupted IFS of the PCC and vPCC may in part explain the impaired cognitive and executive function of AD patients. The finding of specific disrupted regions may provide evidence for early accurate diagnosis and clues for future targeted treatment.

### Limitations

There are several limitations in the present work. First, this study included a small sample size. Second, our research focused only on cross-sectional data, which did not allow us to track the specific alterations in the causal interactions among inter-system during different stages of the disease. Third, we did not perform related behavioral tests. Specific cognitive and executive function impairment in AD patients may be related to changes in the IFS of the inter-systems. Considering these limitations, future work should involve a longitudinal study [including different stages of AD, i.e., from mild cognitive impairment (MCI) to mild, moderate, and severe AD] to investigate the sequence of the changes in different systems; we will recruit more participants and collect multi-center imaging data to enhance the stability of the results. In addition, we should explore the relationships between the changes in the causal interactions among the inter-systems and specific cognitive and executive abilities.

## Conclusions

To summarize, significant disruption of the DN itself was found to be closely related to the changes in its interactions with other systems in AD patients. Distinct DN systems showed different patterns of disruption and/or incoordination in their causal interactions with the FPCN subsystems and DAN. Additionally, the DN subsystems also play a crucial role in the disordered temporal co-evolution of sets of inter-system interactions in AD. Furthermore, the FPCNA had a higher regulatory burden on the Core; the MTL subsystem and Core contributed the most to the classification between the AD and NCs. These findings indicated that disrupted DN subsystems initiated disrupted and disordered interactions with the FPCNA/B and DAN and caused a higher regulatory burden of FPCNA. A detailed exploration of the changes in the causal interactions among these large-scale network subsystems and the initial elements that caused these alterations may be helpful for further understanding the specific system-level pathogenesis of AD, might provide a potential imaging biomarker for the early accurate diagnosis of AD, and find clues for therapeutic interventions targeting large-scale brain networks in AD.

## Data Availability Statement

The raw data supporting the conclusions of this article will be made available by the authors, without undue reservation.

## Ethics Statement

The studies involving human participants were reviewed and approved by Ethics Review Committee of Huashan Hospital affiliated to Fudan University. The patients/participants provided their written informed consent to participate in this study. Written informed consent was obtained from the individual(s) for the publication of any potentially identifiable images or data included in this article.

## Author Contributions

HQ and PW designed the study. HQ and YH analyzed the data and revised the manuscript. HQ interpreted the data and drafted the manuscript. YL acquired the data. All authors read and approved the final manuscript.

## Conflict of Interest

The authors declare that the research was conducted in the absence of any commercial or financial relationships that could be construed as a potential conflict of interest.

## Abbreviations

FPCN: frontoparietal control network
DN: default network
DM: dorsomedial prefrontal cortex
MTL: medial temporal lobe
DAN: dorsal attention network
LTC: lateral temporal cortex
PCC: posterior cingulate cortex
vPCC: ventral posterior cingulate cortex
pIPL: posterior inferior parietal lobule
RMPFC: rostromedial prefrontal cortex
DM: dorsomedial prefrontal cortex
pDLPFC: posterior dorsolateral prefrontal cortex
TP: temporopolar cortex
PrCv: ventral precentral cortex
aMT: anterior middle temporal region
pIFS: posterior inferior frontal sulcus
aIFS: anterior inferior frontal sulcus
aIPL: anterior inferior parietal lobule
IFS: information flow strength
rs-fMRI: Resting-state functional magnetic resonance imaging
BOLD: blood oxygen level-dependent
L: left hemisphere
R: right hemisphere
SVM: support vector machine
LOOCV: leave-one-out cross-validation.
